# Neurotoxicological profile of the hallucinogenic compound 25I-NBOMe

**DOI:** 10.1038/s41598-022-07069-8

**Published:** 2022-02-21

**Authors:** Monika Herian, Adam Wojtas, Marzena Maćkowiak, Agnieszka Wawrzczak-Bargiela, Anna Solarz, Agnieszka Bysiek, Katarzyna Madej, Krystyna Gołembiowska

**Affiliations:** 1 Department of Pharmacology, Maj Institute of Pharmacology, Polish Academy of Sciences, 12 Smętna, 31-343 Kraków, Poland; 2 Laboratory of Pharmacology and Brain Biostructure, Department of Pharmacology, Maj Institute of Pharmacology, Polish Academy of Sciences, 12 Smętna, 31-343 Kraków, Poland; 3 Department of Analytical Chemistry, Faculty of Chemistry, Jagiellonian University, 2 Gronostajowa, 30-387 Kraków, Poland

**Keywords:** Cell biology, Neuroscience

## Abstract

4-Iodo-2,5-dimethoxy-*N*-(2-methoxybenzyl)phenethylamine (25I-NBOMe) is a new psychoactive substance with strong hallucinogenic properties. Our previous data reported increased release of dopamine, serotonin, and glutamate after acute injections and a tolerance development in the neurotransmitters release and rats’ behavior after chronic treatment with 25I-NBOMe. The recreational use of 25I-NBOMe is associated with severe intoxication and deaths in humans. There is no data about 25I-NBOMe in vivo toxicity towards the brain tissue. In this article 25I-NBOMe-crossing through the blood–brain barrier (BBB), the impact on DNA damage, apoptosis induction, and changes in the number of cortical and hippocampal cells were studied. The presence of 25I-NBOMe in several brain regions shortly after the drug administration and its accumulation after multiple injections was found. The DNA damage was detected 72 h after the chronic treatment. On the contrary, at the same time point apoptotic signal was not identified. A decrease in the number of glial but not in neural cells in the frontal (FC) and medial prefrontal cortex (mPFC) was observed. The obtained data indicate that 25I-NBOMe passes easily across the BBB and accumulates in the brain tissue. Observed oxidative DNA damage may lead to the glial cells’ death.

## Introduction

From the physiological point of view, hallucinogens are one of the safest classes of psychoactive substances. They powerfully alter perception and mood but do not produce dependence and addiction^1^. The most popular classic psychedelics representative, i.e., lysergic acid diethylamide (LSD), does not seem to evoke direct toxicity in humans^2^. However, hallucinogens belonging to the so-called new psychoactive substances (NPS) are related to many severe intoxications and deaths^3^. In 2003, phenethylamine derivatives from the NBOMe series were synthesized^4^ for research purposes as potent serotonin 5-HT_2A_ receptor agonists, and in 2010 they appeared on the illicit drug market^5^. NBOMe compounds are *N*-2-methoxy-benzyl substituted 2C hallucinogens that exhibit a nanomolar binding affinity mainly to the serotonin 5-HT_2A_ (0.5–1.6 nM) and 5-HT_2C_ (4.6–130 nM) receptors^6^. Up to date studies confirmed the impact of 25I-NBOMe on several neurotransmitters’ pathways. The drug affects the dopamine system pointing out its possible addictive properties^7^. What is more, 25I-NBOMe impacts the serotonin system and causes the characteristic for hallucinogens to increase in the cortical glutamate release^8^. Our recent study confirmed the contribution of 5-HT_2A_ and 5-HT_2C_ receptors in effect on both brain neurotransmission and hallucinogenic activity in rats^9^. Moreover, the tolerance development in the neurotransmitters’ release and animals behavior was observed after 7-days treatment with 25I-NBOMe^10^. Due to their similar pharmacological effects on a human, NBOMe series were sold as LSD^11^. Available case studies confirm NBOMe-linked toxicities at low doses, with the dose of 50 μg being sufficient to produce psychoactive effects. 4-Iodo-2,5-dimethoxy-*N*-(2-methoxybenzyl)phenethylamine (25I-NBOMe), the most commonly encountered of the NBOMe group, has been identified in plasma, urine, and postmortem heart blood. Typical physiological effects after NBOMe intake include hypertension, tachycardia, diaphoresis, and dilated pupils. Moreover, NBOMe toxidrome, apart from hallucination induction, also causes neuropsychiatric effects, such as delirium, agitation, aggression, seizures, and perceptual disturbances^5^. According to some reports, the occurrence of NBOMe compounds was confirmed in the same blotters marked as LSD. Moreover, NBOMe series were found in tablets sold as MDMA^12^. Injections with 25I-NBOMe (encoded as 25I-NBMeO) at doses 0.1, 0.3, and 1 mg/kg significantly decreased the number of surviving BrdU+ cells and BrdU/NeuN+ neurons in the hippocampus in comparison to control rats suggesting decreased neurogenesis in this brain region^13^. In the in vitro studies, 25I-NBOMe decreased the viability of H9c2 cells and primary mice cardiomyocytes. Moreover, in vitro activity of p21 (CDC42/RAC)-activated kinase 1 (PAK1) was also reduced, and the QT interval measured by electrocardiography in rats was prolonged. Thus, it may be stated that the drug produces cardiotoxicity^14^. Furthermore, in vitro studies with a close congener of this class, 25C-NBOMe (25–400 µM), showed a potent reduction of SN4741, SH-SY5Y, and PC12 cells viability indicating 50 times higher potency to reduce SH-SY5Y cells in respect to methamphetamine^15^. The incubation of the rat primary cortical cultures with 25B-NBOMe (30 μM) decreased neuronal activity that did not recover after 19 h of washout period^16^. Our in vivo studies indicated that the low dose of 25B-NBOMe (0.3 mg/kg) was potent in damaging DNA in the rat frontal cortex^17^.

Although many studies report adverse effects like seizures^18^, intoxications, and deaths, aspects of the in vivo 25I-NBOMe-mechanism of neurotoxicity has to be explored in depth. Thus, the present study aimed to assess the induction of in vivo neurotoxicity in the rat brain after single and repeated 25I-NBOMe treatment. The distribution of the drug in brain regions was determined using mass spectrometry. DNA damage of 25I-NBOMe was evaluated using a comet assay test, and the apoptotic signal was detected using TUNEL assay. Moreover, an immunohistochemical assessment of cells number in the rat brain was studied.

## Results

### 25I-NBOMe distribution in the rat blood plasma and brain regions

**Figure 1:**
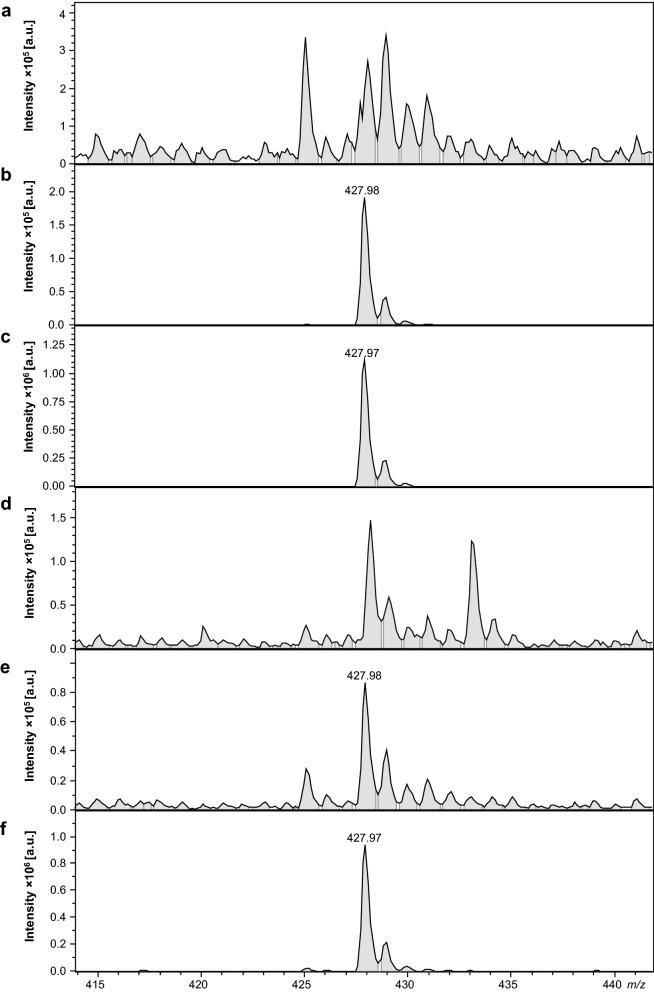
Mass spectra of the blood serum: control (**a**), 25I-NBOMe 1 mg/kg (**b**), 25I-NBOMe 10 mg/kg (**c**) and the frontal cortex analysis: control (**d**), 25I-NBOMe 1 mg/kg (**e**), 25I-NBOMe 10 mg/kg (**f**), indicating the occurrence of the 25I-NBOMe precursor ion (~ 428 m/z); *a.u.* arbitrary units.

**Figure 2:**
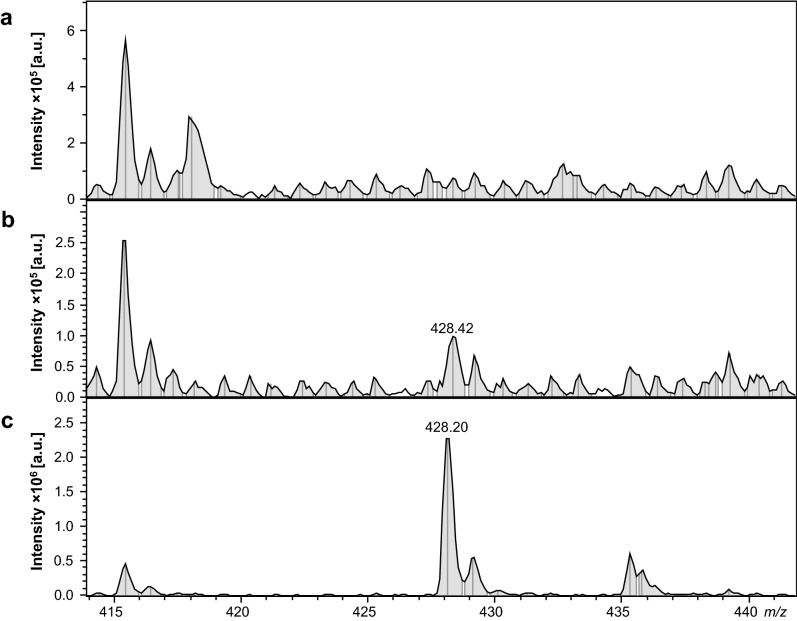
Mass spectra of the frontal cortex: control (**a**), 25I-NBOMe 0.3 mg/kg (**b**), 25I-NBOMe 0.3 mg/kg × 7 (**c**), indicating the occurrence of the 25I-NBOMe precursor ion (~ 428 m/z); *a.u.* arbitrary units.

The performed analysis confirmed the presence of 25I-NBOMe with the precursor 428.14 m/z ion and two fragmentation ions 121.62 and 272.28 m/z (Supplementary, Fig. 1S). The drug was detected in the rat blood plasma and brain regions 15 min after 1 and 10 mg/kg single doses of 25I-NBOMe (Table 1). The most profound intensity of 25I-NBOMe precursor ion was found in the nucleus accumbens. A signal dose-dependence was observed in both the blood plasma sample and the frontal cortex, hippocampus, striatum, and nucleus accumbens. Representative spectral images of LC–MS/MS analyses from the blood plasma and frontal cortex are shown in Fig. 1. 25I-NBOMe presence was observed after single and chronic administration with 0.3 mg/kg 25I-NBOMe (Table 2). However, the signal intensity after 7-days treatment in all studied brain regions was higher in comparison to a single dose. The highest intensity after repeated injections was observed in the frontal cortex (Fig. 2).

**Table 1:** Intensity of the 25I-NBOMe precursor ion (~ 428 m*/*z) from one sample preparation. The analysis was performed 15 min after injection with a single low and high (1 and 10 mg/kg) dose of 25I-NBOMe. The values are presented as intensity per 1 mg of wet tissue or 1 ml of blood serum; *a.u.* arbitrary units.

Biological material	Experimental group	Intensity [a.u.]
Blood plasma	Control	7.05 × 10^2^
	1 mg/kg	9.24 × 10^4^
	10 mg/kg	5.44 × 10^5^
Frontal cortex	Control	5.72 × 10^2^
	1 mg/kg	2.94 × 10^4^
	10 mg/kg	4.36 × 10^5^
Hippocampus	Control	1.22 × 10^3^
	1 mg/kg	1.60 × 10^4^
	10 mg/kg	2.71 × 10^5^
Striatum	Control	3.14 × 10^2^
	1 mg/kg	5.97 × 10^4^
	10 mg/kg	6.95 × 10^5^
Nucleus accumbens	Control	1.17 × 10^2^
	1 mg/kg	2.32 × 10^7^
	10 mg/kg	7.15 × 10^7^

**Table 2:** Intensity of the 25I-NBOMe precursor ion (~ 428 m/z) from one sample preparation. The analysis was performed 72 h after a single and chronic injection (0.3 or 0.3 mg/kg × 7) with 25I-NBOMe. Values are presented as intensity per 1 mg of wet tissue; *a.u.* arbitrary units.

Biological material	Experimental group	Intensity [a.u.]
Frontal cortex	Control	3.29 × 10^2^
	0.3 mg/kg	2.25 × 10^3^
	0.3 mg/kg × 7	2.93 × 10^4^
Hippocampus	Control	2.46 × 10^2^
	0.3 mg/kg	6.89 × 10^3^
	0.3 mg/kg × 7	1.72 × 10^4^
Striatum	Control	2.51 × 10^2^
	0.3 mg/kg	1.61 × 10^3^
	0.3 mg/kg × 7	2.12 × 10^3^
Nucleus accumbens	Control	3.02 × 10^2^
	0.3 mg/kg	3.85 × 10^3^
	0.3 mg/kg × 7	9.00 × 10^3^

### Cortical and hippocampal cells number after 25I-NBOMe administration

**Figure 3:**
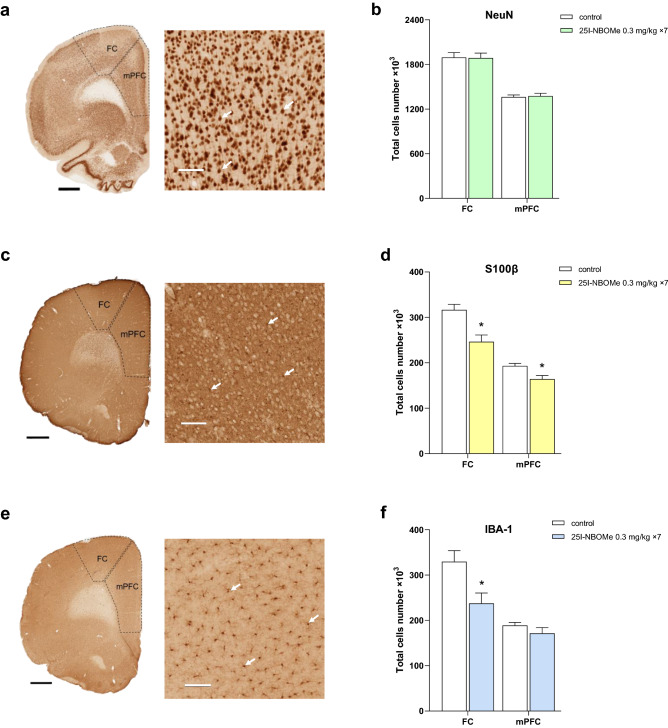
The number of neurons and glia cells in the frontal cortex (FC) and medial prefrontal cortex (mPFC) (n = 6 per group). Light photomicrographs that were immunoprobed for NeuN (**a**), S100β (**c**), IBA-1 (**e**) and examined for the number of immunopositive neurons (**b**), astrocytes (**d**), and microglia (**f**). The graph bars indicate the mean ± SEM. The scale bars represent 1 mm and 100 μm. The arrows show examples of cells immunopositive for NeuN, S100β and IBA-1.

**Figure 4:**
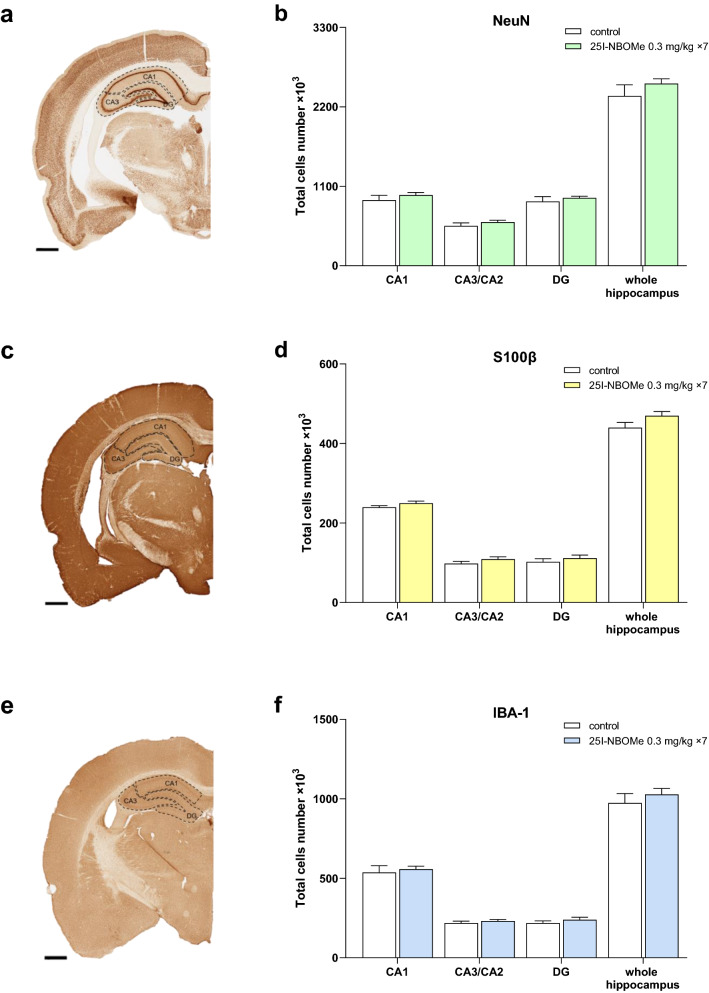
The number of neurons and glia cells in the subregions and in the entire hippocampus (n = 6 per group). The light photomicrographs that were immunoprobed for NeuN (**a**), S100β (**c**), IBA-1 (**e**) and examined for the number of immunopositive neurons (**b**), astrocytes (**d**), and microglia (**f**). The graph bars indicate the mean ± SEM. The scale bars represent 1 mm; *DG* dentate gyrus.

The usage of antibodies specifically recognizing neurons, astrocytes, and microglia allowed the assessment of the changes in the immunopositive cells number in the medial prefrontal cortex (mPFC), frontal cortex (FC), and hippocampus (Figs. 3 and 4, respectively). The number of S100β- (F_1,10_ = 13.2, p < 0.01) and IBA-1-immunopositive cells (F_1,10_ = 7.6, p < 0.02) was significantly decreased in the FC (Fig. 3d,f). The S100β-immunopositive cells significant decrease (F_1,10_ = 9.0, p < 0.01) was observed in the mPFC of animals treated chronically with 25I-NBOMe (Fig. 3d). We did not notice any significant change in the number of NeuN cells in the FC (F_1,10_ = 0.01, p < 0.90) and mPFC (F_1,10_ = 0.08, p < 0.78) (Fig. 3b). On the other hand, immunohistochemical staining did not show any changes in the number of neuronal and glial cells in all analyzed hippocampal regions: CA1, CA3/CA2, and dentate gyrus (DG) (Fig. 4b,d,f). The most enriched in all types of cells i.e., neurons, astrocytes, and microglia, were hippocampal CA1 and DG regions.

### The effect of 25I-NBOMe on DNA damages in the rat frontal cortex and hippocampus

**Figure 5:**
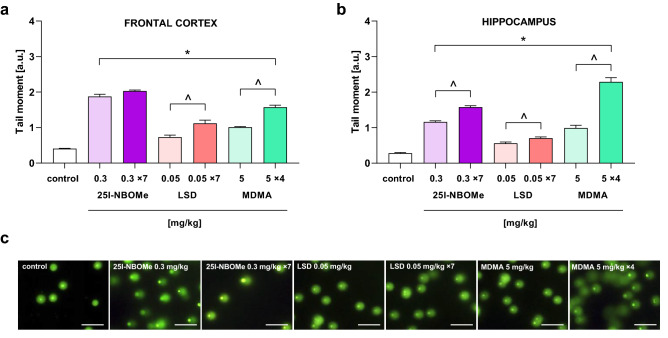
The effect of a single and repeated doses of 25I-NBOMe (0.3 mg/kg), LSD (0.05 mg/kg) and MDMA (5 mg/kg) on the oxidative damage of DNA in the nuclei from rat frontal cortex (that consist of the sum of FC and mPFC; **a**) and hippocampus (**b**). Data are the mean ± SEM (n = 6–13 per group) and represent tail moment shown as the product of the tail length and the fraction of total DNA in the tail. (**c**) Typical microscopic images of nuclei from control, 25I-NBOMe, LSD and MDMA treated rats (scale bars = 100 μm); *p < 0.05 in comparison to control group; ^^^p < 0.02 acute vs. chronic; *a.u.* arbitrary units.

Single and repeated treatment with 0.3 mg/kg of 25I-NBOMe produced DNA damage by reactive oxygen species (ROS) in the rat frontal cortex (F_2,29_ = 237, p < 0.0001) and hippocampus (F_2,23_ = 275, p < 0.0001), presented as a percent of tail moment measured at 72 h after the drug administration (Fig. 5a,b, respectively). Only in the hippocampus was the DNA damage observed after multiple injections stronger than after a single dose of 25I-NBOMe (p < 0.01). LSD, as a comparative compound, caused DNA damage in the frontal cortex after a single and a 7-day treatment at the dose 0.05 mg/kg in comparison to control, and this damage was significantly greater after chronic administration (F_2,20_ = 22, p < 0.0001). There was a significant increase in the tail moment value in the hippocampus after acute and chronic treatment with LSD (F_2,17_ = 42, p < 0.0001). Similarly to the frontal cortex, the repeated injections caused a higher DNA damage than the single dose (p < 0.02). MDMA, as a positive control in this test, given acutely and chronically at the dose of 5 mg/kg exhibited DNA damaging properties in the frontal cortex (F_2,19_ = 123, p < 0.0001) and hippocampus (F_2,17_ = 149, p < 0.0001). There was a stronger increase in the tail moment value in the frontal cortex (p < 0.001) and hippocampus (p < 0.001) after four injections of MDMA in comparison to the acute one. Typical microscopic images of nuclei from control, 25I-NBOMe, LSD and MDMA-treated rats were shown in Fig. 5c.

### Apoptotic signal in the rat frontal cortex and hippocampus after 25I-NBOMe administration

**Figure 6:**
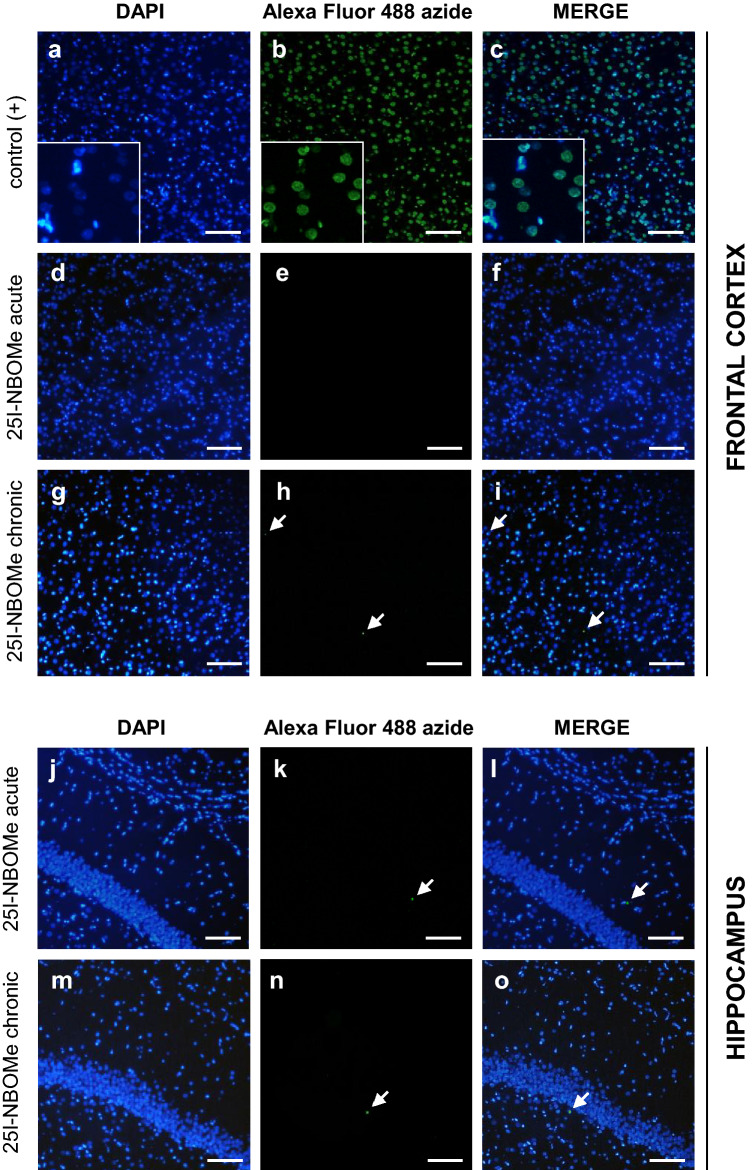
Apoptosis measured by TUNEL assay in rats treated with a single (0.3 mg/kg; **d**–**f**, **j**–**l**) or repeated (0.3 mg/kg × 7; **g**–**i**, **m**–**o**) dose of 25I-NBOMe (n = 4) in the rat frontal cortex and hippocampus. The brain slices treated with DNAse being a positive control are shown in a, b, and c. The arrows show apoptotic nuclei indicated by green (**b**,**e**,**h**,**k**,**n**). Blue spots represent nuclei stained by DAPI (**a**,**d**,**g**,**j**,**m**), and merged images are shown in (**c**,**f**,**i**,**l**,**o**) (scale bars = 100 μm).

In this experiment, we evaluated the effect of 25I-NBOMe on apoptosis induction 72 h after the treatment. In contrast to a positive control (brain slice damaged with DNAse), the performed assay did not indicate apoptotic signal in brain slices from rats treated acutely or chronically with 25I-NBOMe given at the dose of 0.3 mg/kg. Since qualitative examination of sliced material did not provide any positive signal from the rat frontal cortex and anterior and posterior hippocampus (only a few isolated cells), the quantitative examination was not continued. The representative data are presented in Fig. 6.

## Discussion

Our study indicated that 25I-NBOMe easily crosses the blood–brain barrier (BBB) since it was detected both in the blood serum and several brain regions 15 min after the injection. Repeated administration of 25I-NBOMe caused drug accumulation in the brain tissue. Seven-days treatment with 0.3 mg/kg of 25I-NBOMe decreased the number of astrocytes in the FC and mPFC and the number of microglia cells in the FC. On the other hand, the chronic treatment did not affect the number of cells in the hippocampus. Furthermore, the drug produced double- and single-strand DNA breaks by ROS after single and multiple injections in rats’ frontal cortex and hippocampus. However, these changes did not lead to cell death by apoptosis. Our data, for the first time, presents the in vivo neurotoxicity of 25I-NBOMe.

25I-NBOMe was detected in the blood serum shortly after subcutaneous injections. The calculated spectral intensity was an order of magnitude higher after the 10 mg/kg dose than 1 mg/kg. Similarly, 15 min after injections, the drug was detected in the brain structures, mainly in the nucleus accumbens. It suggests fast and efficient penetration of 25I-NBOMe to the brain. The high intensity of 25I-NBOMe spectra from the nucleus accumbens and striatum could be explained by the first pass of the drug through more ventral subcortical brain regions and then crossing to more distant dorsal regions, such as cortex and hippocampus. Repeated administration with 0.3 mg/kg for 7 days of 25I-NBOMe resulted in the drug accumulation in all studied brain regions since the precursor ion intensity was increased in this experimental group compared to the single-injected one. In Ettrup et al.^19^ study, carbon-11 labelled 25I-NBOMe known as ^11^C-CIMBI-5 was located in the cortex and the lower range in the cerebellum shortly after the intravenous injection. In our study, among other brain regions, 25I-NBOMe accumulated mostly in the frontal cortex and hippocampus. This effect may be explained by the significant density of the 5-HT_2A_ receptors to which 25I-NBOMe exhibits a high binding affinity and which are located on both cortical pyramidal cells and GABA interneurons^20,21^ as well as pyramidal cells in CA1, CA2, and CA3 regions of the hippocampus^22^.

In vitro studies with the NBOMe series suggest toxic properties of these drugs. Twenty-four hour incubation with a wide range of 25C-NBOMe doses potently reduced the viability of cells^15^. Another congener 25B-NBOMe was also cytotoxic and attenuated neuronal activity of primary cortical cultures^16^. Our in vivo experiments indicated astrocytic cells loss as labeled with S100β after repeated treatment with 25I-NBOMe in the FC and mPFC and decreased the number of microglia cells as labeled with IBA-1 in the FC. Presented results do not indicate neurons loss in both cortical regions and the rats' hippocampus. S100β is a monomeric protein primarily synthesized by the end feet process of the astrocytes. It was found mainly in the nucleus and cytoplasm of astrocytes. S100β released from the cell may have trophic or toxic effects depending on its concentration. It has a neuroprotective effect at low concentrations, while at higher doses, it inhibits neuronal proliferation and differentiation and induces neurodegeneration and apoptosis^23^. In our immunohistochemical studies, we observed a lower number of S100β-positive cells in the FC and mPFC that may correspond to a decreased number of astrocytes in response to 25I-NBOMe treatment. Furthermore, astrocytes may release S100β protein extracellularly, affecting both astrocytes itself and neurons^24^. It is believed that a high S100β protein level is observed in a wide variety of pathological conditions and is negatively correlated with BBB integrity^25^. On the other hand, IBA-1 is a cytoplasmic protein specifically expressed in microglia. The role of microglia is mainly related to phagocytosis and removing damaged and apoptotic cells, neurofibrillary tangles, and plaques, but also DNA fragments^26^. Moreover, apart from the phagocytosis of apoptotic cells, microglia are also a key molecules in proinflammatory activity, maintaining homeostasis and controlling the fate of neurons and their progenitors^27^. It could be speculated that decreased number of microglia and astrocytes observed in our experiments may be related to the weakened repair mechanisms and defense functions of these cell types. Thus, 25I-NBOMe may pass easily and quickly through the damaged BBB and accumulate after chronic administration in the brain tissue, as demonstrated in our study.

Observed DNA damage at 72 h after single and repeated administration suggests neurotoxic properties of 25I-NBOMe. Cortical cells seem to be less resistant to the influence of 25I-NBOMe since our immunohistochemical studies indicated changes in cells number only in the FC and mPFC but not in the hippocampus. What is more, mass spectrometry analyses showed a higher accumulation of the drug in the frontal cortex with respect to the hippocampus. The in vitro work of Cocchi et al.^28^ suggests the genotoxic capacity of phenethylamines in high concentration and hypothesizes a possible involvement of oxidative stress that does not always minimize cell viability. Our data is in vivo confirmation of this study showing the DNA damaging effect of phenethylamine congener 25I-NBOMe. Furthermore, our findings show the regional difference in neurotoxicity of 25I-NBOMe, indicating its damaging impact on cortical microglia and astrocytes being the important defense line in the brain. The lack of apoptotic signal detection in the TUNEL assay may be related to the time point of 72 h as insufficient to induce programmed cell death. For better understanding of a time of the apoptosis induction, the research in different time points is needed. Nevertheless, our data indicate the decrease in markers of glial cells, which corresponds to the appearance of double- and single-strand DNA breaks and points to the defective defense system of these cells but not neurons. Notably, the difference between brain regions in cells resistance against damage is noticed. Interestingly, LSD was weaker in its damaging effect on DNA than 25I-NBOMe due to its different receptor profiles^6^ and its different impact on glutamatergic and dopaminergic neurotransmission^29^. Although, LSD is considered to be a safe substance, there are some older reports pointing out the possibility of causing chromosomal aberrations in vitro and in vivo, especially in the higher doses and in the long-term use^30,31^. Moreover, it was proposed that LSD may act directly on DNA by intercalation within DNA helix producing conformational changes that were not sufficient to decrease internal stability^32^. Thus, our data are the first that assess LSD effect on DNA damage studied in the comet assay. MDMA, known as oxidative stress inductor^33,34^ and used in our study as a comparator, was nearly equal to 25I-NBOMe in oxidative damage of DNA in both brain regions.

## Conclusion

Our data clearly indicate that phenethylamine hallucinogen 25I-NBOMe sold as a replacement for LSD and acting with similar potency as LSD may cause severe neurotoxicity by inducing oxidative stress and suppressing the defense role of astrocytic and microglial cells in rat frontal cortex and hippocampus.

## Materials and methods

### Animals

All experiments were performed on male Wistar–Han rats (Charles River, Sulzfeld, Germany), weighting 280–320 g. The animals were initially acclimatized and housed in groups of 5 each in temperature (23 ± 1 °C) and humidity (55 ± 10%) controlled rooms under a 12 h light/dark cycle (light was turned on at 6 a.m.), with free access to tap water and standard laboratory food (VRF 1, Special Diets Services, Witham, UK). The experiments were conducted in accordance with the European regulations for animal experimentation (EU Directive 2010/63/EU on the protection of animals used for scientific purposes). The 2nd Local Institutional Animal Care and Use Committee (IACUC) in Kraków, Poland (Permit number: 189/2017) approved the experimental protocols. The study was carried out in compliance with the ARRIVE guidelines. This article does not contain any studies with human participants by any of the Authors.

### Drugs and reagents

4-Iodo-2,5-dimethoxy-*N*-(2-methoxybenzyl)phenethylamine hydrochloride (25I-NBOMe) was purchased from Chiron AS (Trondheim, Norway). The chemicals used for the alkaline comet assay were from Trevigen (Gaithersburg, MD, USA) and Merck (Warszawa, Poland). TUNEL assay was performed with the usage of Click-iT™ Plus TUNEL Assay of Invitrogen, Thermo Fisher Scientific (Waltham, MA, USA). The reagents used in immunohistochemistry came from Sigma Aldrich (Poznań, Poland), Vector Laboratories (Burlingame, CA, USA), and Proteintech (Manchester, UK) and the one for mass spectrometry were from Merck (Warszawa, Poland). Animals were anesthetized with the usage of sodium pentobarbital (Biowet Puławy, Poland).

### Drug administration

Animals received single or multiple (once per day for seven days) subcutaneous injections of 25I-NBOMe dissolved in 0.9% NaCl at a dose of 0.3 mg/kg/2 ml. The control group was treated with 0.9% NaCl solution in the same way. All procedures were performed 72 h after the last injection. The time point of 72 h was chosen due to the possible generation of neurotoxicity via oxidative stress caused by reactive oxygen species (ROS). Exceptionally, for initial 25I-NBOMe brain distribution studies, rats received the drug in the dose of 1 and 10 mg/kg and were sacrificed 15 min after the drug administration. The rat blood plasma and brain tissues were collected immediately after rats’ decapitation. LSD (0.05 mg/kg, ip) was given daily for 7 days in a volume of 1 ml/kg, while MDMA 5 mg/kg, ip was injected in a volume of 2 ml/kg every second day (overall 4 injections).

### LC–MS/MS with electrospray ion trap mass spectrometry (ESI–MS)

The presence of 25I-NBOMe in the rat brain regions and blood serum was analyzed with electrospray ion trap mass spectrometry (Amazon SL from Bruker Daltonics, Bremen, Germany). At 72 h after the last injection with 25I-NBOMe or 15 min after injection of single low and high (1 and 10 mg/kg) dose, animals were sacrificed by decapitation. Brains were removed and the frontal cortex, striatum, nucleus accumbens, and hippocampus were dissected. The blood samples were also collected after 25I-NBOMe injections. Tissue samples were homogenized in an ice-cold 0.1 M HClO_4_, and were centrifuged at 10,000×*g* for 10 min at 4 °C. Blood was incubated for 30 min and then centrifuged for 15 min, 3000 rpm at 4 °C. The collected blood serum and tissue supernatants were frozen at − 80 °C until the time of mass spectrometry analysis. The value pH of samples was about 2. To purify the samples, solid phase extraction (SPE) columns MacroSpin Columns Silica C18 (The Nest Group, Inc., Southborough, USA) were used. Initially, columns were activated with 100% methanol (MeOH) solution by double centrifugation at 110×*g* for 1 min. Next, to remove MeOH, 2% acetonitrile (ACN) with 0.1% HCOOH was used. Subsequently, the columns were washed twice with the blood and tissue samples. To remove adenosine-5'-diphosphate (ADP) that has the same precursor ion as 25I-NBOMe the columns were washed twice with 2% ACN. Finally, to wash out 25I-NBOMe, the column was washed twice with 80% ACN with 0.1% HCOOH. Samples were left to dry in SpeedVac Vacuum Concentrator (Labconco, Kansas City, USA) overnight. The following day, samples were dissolved in 10% ACN with 0.1% HCOOH and injected to the LC–MS/MS system. The nanoLC-MS/MS analysis used to separate compounds was performed using the Ultimate 3000 nanocapillary chromatography system (Polygen, Gliwice, Poland). The separations were performed using a PepMap column (15 cm long, 75 μm ID, C18, 3 μm particle size, 100 Å pore size, Thermo-Scientific). The gradient was formed using 0.1% HCOOH in H_2_O (solvent A) and 0.1% HCOOH in ACN (solvent B), at a flow rate of 300 nl/min. The system was controlled by the Hystar software (Bruker Daltonics). A gradient was produced from 5 to 80% B in 100 min, then kept at 90% of phase B for 10 min. The mass spectrometry detection was performed in positive ion mode by electrospray ion trap mass spectrometry (Amazon SL from Bruker Daltonics, Bremen, Germany). The retention time of 25I-NBOMe was about 30.3 min. The acquired mass spectra were analyzed using the Bruker Data Analysis v. 4.0 software (Bruker Daltonics). The data presented in tables are calculated as intensity per 1 mg of wet tissue or 1 ml of blood serum, while mass spectra represent the real intensity of 25I-NBOMe-precursor ion from tissue samples with different mass.

### Immunohistochemistry

Animals were deeply anesthetized and transcardially perfused with 0.9% NaCl followed by 4% paraformaldehyde (PFA) in 0.1 M phosphate-buffered saline (PBS). After 24 h of fixation in 4% PFA (4 °C), 300 µm sections were cut through the frontal cortex (FC) and medial prefrontal cortex (mPFC) as well as hippocampus using a VT-1000S vibratome (Leica Microsystems, Heidelberg, Germany). Free-floating sections were processed for single staining of neurons with neuronal-specific nuclear protein (NeuN), glia with specific calcium-binding protein B (S100β), and microglia cells with ionized calcium-binding adaptor molecule 1 (IBA-1) antibodies. Subsequently, brain sections were rinsed and incubated for 1 h in a blocking buffer: 0.01 M PBS containing 0.3% Triton X-100 and 5% average horse, goat, or rabbit serum. After that, the sections were incubated for 48 h at 4 °C with one of the following primary antibodies: monoclonal anti-NeuN (1:1000), monoclonal anti-S100β-subunit (1:1000) or polyclonal IBA-1 (1:500) diluted in 0.01 M PBS containing 0.3% Triton X-100 (PBST) and 3% average horse or goat serum. Primary antibody binding was visualized with biotinylated secondary antibodies, the Avidin/Biotin Complex (Vectastain Elite ABC Kit) according to recommended by manufacturer concentration and 3,3′-diaminobenzidine tetrahydrochloride (DAB, 10 mg/50 ml and 0.025% H_2_O_2_) solution to give a brown color to NeuN, S100β and IBA-1-immunoreactive cells. For data presentation, digital images were captured using a digital camera CX 9000 (Bioscience Microbrightfield, Inc., Germany) attached to a Leica microscope (CTR 6000) with 2.5 and 5.0 dry or 63 × and 100 × oil objectives (Leica) that was controlled by Stereo Investigator software (v.8.10.2, 1995–2007 Bioscience Microbrightfield, Inc., Germany). The final photomicrographs were composed using the Adobe Photoshop program. The numbers of NeuN-, S100β- and IBA-1-immunopositive cells in the analyzed brain regions were estimated using unbiased stereological methods^35,36^. Briefly, every sixth section from the systematic random sampling along the rostrocaudal axis was analyzed with a 63 ×/1.4–0.7 lens using the Stereo Investigator stereology system software. The cells appearing in the upper focal plane were omitted to prevent the counting of cell caps (− 5 μm of the topmost surface of the section). Immunopositive cells were marked when they were within the optical dissectors, which comprised a focal plane of 1600 μm^2^ × 15 μm. The total numbers of NeuN, S100β- and IBA-1-immunopositive cells in the FC, mPFC, and hippocampus were automatically calculated by the Stereo Investigator software. In addition, the volume of the cortical regions and hippocampus was calculated using the Cavalieri Estimator option of the Stereo Investigator software.

### Alkaline comet assay

The alkaline comet assay was performed with the use of CometAssay^®^ Reagent Kit for Single Cell Gel Electrophoresis Assay. At 72 h after the acute and chronic treatment with 25I-NBOMe (0.3 mg/kg, sc × 7 days), LSD (0.05 mg/kg, ip × 7 days) or MDMA (5 mg/kg, ip × 4 doses every second day) animals were sacrificed by decapitation and the frontal cortex (that consist of the sum of FC and mPFC) and hippocampus were dissected. After homogenization and several purification and centrifugation stages (as described previously in^17^), the nuclear suspension was obtained using a sucrose gradient (2.8 M/2.6 M, bottom to top). The nuclear fraction was mixed with low melting point agarose and transferred immediately onto CometSlides™. The following steps, including membrane lysis, DNA unwinding, alkaline electrophoresis, and staining (SYBR^®^ Gold), were carried out according to Trevigen CometAssay^®^ protocol. Stained sections were acquired and analyzed under a fluorescence microscope (Nikon Eclipse50i, Japan) equipped with a camera and NIS Elements software. The data was analyzed using OpenComet software v.1.3 (cometbio.org), a plugin of ImageJ program v.1.47 (NIH, Bethesda, MD, USA). DNA damage was presented as a tail moment. Tail moment incorporates a measure of both the smallest detectable size of migrating DNA (reflected by the comet tail length) and the number of damaged pieces (represented by the intensity of DNA in the tail).

### Terminal deoxynucleotidyl transferase dUTP nick end labeling (TUNEL) assay

As described above, animals were perfused transcardially and brains were fixed overnight in 4% PFA, then gradually dehydrated and stored in 70% ethanol (EtOH). Paraffin-embedded brain tissue was sectioned on a microtome (Leica, RM45) for 7-µm-thick sections. For further experiments following sections from the region of the frontal cortex, anterior and posterior hippocampus were collected. Next, chosen microscope slides with tissue sections were deparaffinized in xylene and declining concentrations of EtOH and remained in 0.01 M PBS. The apoptotic signal was assessed using Click-iT™ Plus TUNEL Assay for in situ apoptosis detection with Alexa Fluor™ 488 picolyl azide dye. For positive control, brain slices were incubated with 1 unit of DNAse I (A&A Biotechnology, Gdansk, Poland) as recommended by manufacturer. Vectashield Mounting Medium (Vector Laboratories) with DAPI (4′,6-diamidino-2-phenylindole dihydrochloride) was used for slipping and counterstaining the slides. Stained slides were examined under a fluorescence microscope (Nikon Eclipse50i, Japan) equipped with a camera and NIS Elements software.

### Data analysis

Presented results were analyzed with one-way ANOVA followed by Tukey’s post hoc test. The differences were considered significant if the *p*-value was smaller than 0.05. All statistical analyses were carried out using STATISTICA v.10 StatSoft Inc. 1984–2011 (San Francisco, CA, USA), and the figures were prepared in the GraphPad Prism v.9.00 (GraphPad Software Inc., La Jolla, CA, USA) and Adobe Photoshop program v. 19.0.

## Supplementary Information

Supplementary Figure S1.
