# Supplementary material for: Neurotoxicological profile of the hallucinogenic compound 25I-NBOMe
Source: Sci Rep. 2022 Feb 21;12:2939. doi: 10.1038/s41598-022-07069-8 (PMC8861095; doi:10.1038/s41598-022-07069-8)
Supplement: Supplementary file 1 — Supplementary Figure S1. [file 41598_2022_7069_MOESM1_ESM.doc]

**Supplementary Information**

**Neurotoxicological profile of the hallucinogenic compound 25I-NBOMe**

Herian Monika^1^, Wojtas Adam^1^, Maćkowiak Marzena^2^, Wawrzczak-Bargiela Agnieszka^2^, Solarz Anna^2^, Bysiek Agnieszka^1^, Madej Katarzyna^3^, Gołembiowska Krystyna^1^

^1^Maj Institute of Pharmacology, Polish Academy of Sciences, Department of Pharmacology, 31-343 Kraków, 12 Smętna, Poland

^2^Maj Institute of Pharmacology, Polish Academy of Sciences, Department of Pharmacology, Laboratory of Pharmacology and Brain Biostructure, 31-343 Kraków, 12 Smętna, Poland

^3^Jagiellonian University, Faculty of Chemistry, Department of Analytical Chemistry, 30-387 Kraków, 2 Gronostajowa, Poland

Correspondence: Krystyna Gołembiowska, e-mail: nfgolemb@cyf-kr.edu.pl; phone +48 12

662 32 11; fax +48 12 637 45 00


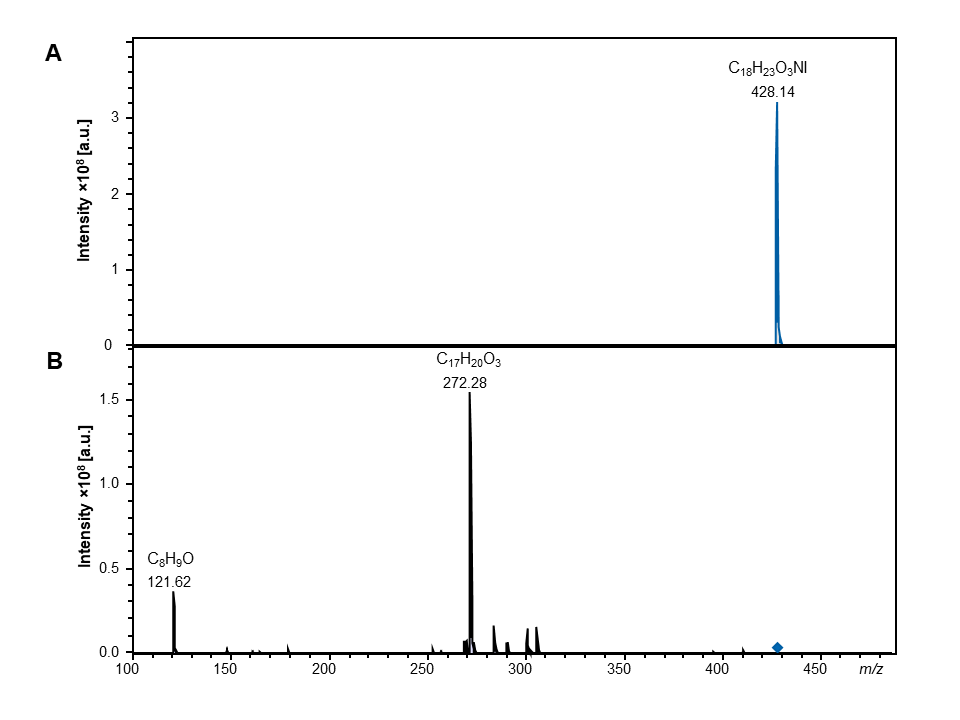


Figure 1S. Mass spectrum (a) and fragmentation (b) spectrum of 25I-NBOMe
